# *LEAF TIP RUMPLED 1* Regulates Leaf Morphology and Salt Tolerance in Rice

**DOI:** 10.3390/ijms23158818

**Published:** 2022-08-08

**Authors:** Jiajia Wang, Yiting Liu, Songping Hu, Jing Xu, Jinqiang Nian, Xiaoping Cao, Minmin Chen, Jiangsu Cen, Xiong Liu, Zhihai Zhang, Dan Liu, Li Zhu, Jiang Hu, Deyong Ren, Zhenyu Gao, Lan Shen, Guojun Dong, Qiang Zhang, Qing Li, Sibin Yu, Qian Qian, Guangheng Zhang

**Affiliations:** 1 State Key Laboratory of Rice Biology, China National Rice Research Institute, Hangzhou 310006, China; 2 National Key Laboratory of Crop Genetic Improvement and National Center of Plant Gene Research, College of Plant Science and Technology, Huazhong Agricultural University, Wuhan 430070, China; 3 Research Center of Plant Functional Genes and Tissue Culture Technology, College of Bioscience and Bioengineering, Jiangxi Agricultural University, Nanchang 330045, China; 4 National Nanfan Research Institute (Sanya), Chinese Academy of Agricultural Sciences, Sanya 572024, China

**Keywords:** *Oryza sativa* L., leaf shape, salt stress, bulliform cells, aquaporin

## Abstract

Leaf morphology is one of the important traits related to ideal plant architecture and is an important factor determining rice stress resistance, which directly affects yield. Wax layers form a barrier to protect plants from different environmental stresses. However, the regulatory effect of wax synthesis genes on leaf morphology and salt tolerance is not well-understood. In this study, we identified a rice mutant, *leaf tip rumpled 1* (*ltr1*), in a mutant library of the classic *japonica* variety Nipponbare. Phenotypic investigation of NPB and *ltr1* suggested that *ltr1* showed rumpled leaf with uneven distribution of bulliform cells and sclerenchyma cells, and disordered vascular bundles. A decrease in seed-setting rate in *ltr1* led to decreased per-plant grain yield. Moreover, *ltr1* was sensitive to salt stress, and *LTR1* was strongly induced by salt stress. Map-based cloning of *LTR1* showed that there was a 2-bp deletion in the eighth exon of *LOC_Os02g40784* in *ltr1*, resulting in a frameshift mutation and early termination of transcription. Subsequently, the candidate gene was confirmed using complementation, overexpression, and knockout analysis of *LOC_Os02g40784*. Functional analysis of *LTR1* showed that it was a wax synthesis gene and constitutively expressed in entire tissues with higher relative expression level in leaves and panicles. Moreover, overexpression of *LTR1* enhanced yield in rice and *LTR1* positively regulates salt stress by affecting water and ion homeostasis. These results lay a theoretical foundation for exploring the molecular mechanism of leaf morphogenesis and stress response, providing a new potential strategy for stress-tolerance breeding.

## 1. Introduction

Leaves are the main photosynthetic organ of plants. Leaf morphology affects the effective photosynthetic area, which affects accumulation of photosynthetic products and subsequent crop yield. In rice, numerous genes associated with leaf morphogenesis have been mined and cloned, such as *SHALLOT-LIKE 1* (*SLL1*) [1], *HOMEODOMAIN CONTAINING PROTEIN4 (OsHB4)* [2], *SEMI-ROLLED LEAF1*(*SRL1*) [3], *Rice outermost cell-specific gene 5 (Roc5)* [4], *AGO1 homologs 1b* (*OsAGO1b*) [5], *Rice outermost cell-specific 8* (*Roc8*) [6], *PHOTO-SENSITIVE LEAF ROLLING 1* (*PSL1*) [7]. These genes regulate leaf morphogenesis through complex interactions among plant hormone signaling pathways, transcription factors, and microRNAs [8,9]. In addition, leaf morphology is also affected by genes associated with ribosomes synthesis, DNA repair, cell cycle process, cuticle development, ion homeostasis, and microtubule arrangement [9]. However, these genes alone are not sufficient to accurately outline the genetic regulatory network of rice leaf morphogenesis in detail. One of the main challenges in modern agriculture is to increase crop yields under different environmental conditions by cultivating ideal plant architecture [10]. Leaf morphology is an important component of plant architecture and improving it contributes to collaborative improvement of stress resistance and yield. In recent years, great progress has been made in the regulation mechanism of leaf morphology and stress resistance. In addition to the key regulatory roles in plant architecture and yield, many genes regulating leaf morphology also affect characteristics such as drought tolerance, nutrient utilization, and disease resistance. For example, *Ideal Plant Architecture 1* (*IPA1*) not only increases rice yield but also improves rice blast resistance, which counters the traditional view that a single gene cannot simultaneously increase yield and disease resistance [11,12,13,14]. *Dwarf 1* (*D1*) is involved in complex network affecting plant height, leaf size, and abiotic stress response [15,16,17]. *PSL1* regulates rice leaf cell wall development and drought tolerance [7]. Higher leaf temperature, respiration rate, lower transpiration rate, and stomatal conductance in *high temperature susceptibility* (*hts*) resulted in high temperature sensitivity of *hts* [18]. Thus, leaf morphology is closely associated with stress resistance, nutrient utilization, disease resistance, and yield.

Soil salinization is an increasingly serious agricultural problem worldwide [19,20], limiting plant growth and crop productivity in saline–alkali areas [20]. Poor irrigation practices, the improper application of fertilizers, and industrial pollution increased soil salinity in cultivated soil, resulting in aggravated soil salinization [21,22]. Most plants had to develop suitable mechanisms to adjust their physiological and biochemical processes to adapt to high salinity environments during their long evolutionary history due to their sessile nature [20]. Significant progresses have been made for salt tolerance mechanism in plants. They developed suitable strategies to regulate ion and osmotic homeostasis and minimize stress damage [23,24], including exclusion of Na^+^ from leaf tissues, compartmentalization of Na^+^ (mainly into vacuoles), and reducing water loss while maximizing water absorption [21,25,26]. However, few favorable genetic loci associated with salt resistance have been identified in the breeding practices of rice. Therefore, breeding potentially yield-penalty-free rice varieties with high salt tolerance is of great significance and an effective way to expand the adaptability and planting area of rice and improve the yield potential of rice in saline–alkali areas.

Wax is the outermost barrier that plays an important role in plant–environment interactions, including plant adaptation to drought environments and various abiotic and biotic stresses. It promotes resistance to ultraviolet (UV) radiation [27] and pests and diseases [28] and protects internal plant tissues from temperature stress [29]. Moreover, the epicuticle wax layer provides the necessary barrier for reducing non-stomatal water loss during drought stress; thereby significantly improve drought tolerance in rice [30,31]. For example, the wax synthesis regulator DROUGHT HYPERSENSITIVE (DHS) interacts with rice outermost cell-specific gene 4 (Roc4), regulating expression of *BODYGUARD* (*BDG*) and thus affecting rice drought tolerance [32,33]. The rice ethylene response factor *WAX SYNTHESIS REGULATORY GENE 1* (*OsWR1*) positively regulates rice wax synthesis and affects drought tolerance by regulating cuticle development and leaf water retention [34]. In addition, wax has a critical effect on the differentiation of plant tissues and organs, such as the morphological development of leaves, fruits, and pollen, thereby affecting plant fertility. Loss function of wax synthesis genes led to morphological abnormalities of flowers and leaves, such as *knb1* (*knobhead*), *bcf1* (*bicentifolia*), and *wax1* in *Arabidopsis* [35]. The *wax2* plants showed disordered leaf structure and fused floral organs in *Arabidopsis* [36]. In rice, most research on wax synthesis genes has focused on pollen development, panicle fertility, and drought resistance; there are few reports on the regulatory role of wax synthesis genes in leaf morphology. Here, we identified a rice mutant *ltr1* with abnormal leaf morphology. This mutant was obtained by ethyl methanesulfonate (EMS) mutagenesis of Nipponbare and was used to isolate and analyze the function of the candidate gene *LTR1* in regulating leaf morphology. We demonstrated that loss function of *LTR1* led to abnormal development of bulliform cells, vascular bundles, and sclerenchyma cells, and to rumpled leaves, decreases in the seed setting rate and yield, and high sensitivity to salt stress. We also confirmed that *LTR1* mediated regulatory activities of aquaporin and ion transporters result in altered water retention and ion homeostasis under salt stress. Hence, function analysis of *LTR1* in leaf morphology and response to salt stress could provide theoretical foundation for molecular mechanism of leaf morphogenesis and salt response in rice and contribute to breeding efforts to develop salt-tolerant varieties with ideal leaf morphology.

## 2. Results

### 2.1. Identification of the ltr1 Mutant

**Figure 1:**
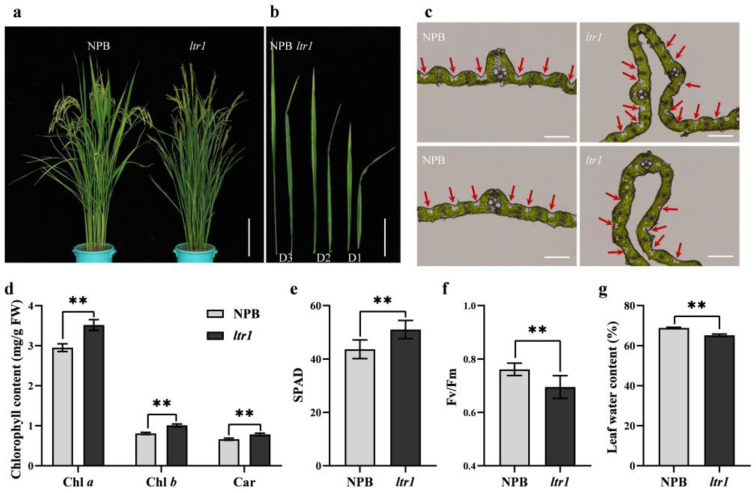
Phenotype analysis of NPB and *ltr1* plants. (**a**) Plant morphology (bar = 20.0 cm), (**b**) leaf morphology (bar = 4.0 cm), and (**c**) observation of frozen sections of NPB and *ltr1* (bar = 200 μm), red arrows in (**c**) represent bulliform cells. (**d**) Chlorophyll content, (**e**) SPAD, (**f**) Fv/Fm, and (**g**) leaf water content of NPB and *ltr1*. Data are given as means ± SD. Asterisks indicate significant difference based on the Student’s *t*-test: ** in the figure represents significant difference at *p* < 0.01.

The *ltr1* mutant was successfully obtained by EMS mutagenesis of the NPB. Phenotypic observation indicated that *ltr1* exhibited abnormal leaf morphology with uneven distribution of bulliform cell on adaxial surface and sclerenchyma cells on abaxial surface and disordered vascular bundles (Figure 1a–c). The contents of chlorophyll *a*, chlorophyll *b*, and carotenoids were significantly higher in *ltr1* than in NPB, with increases of 19.23%, 24.96%, and 17.83%, respectively (Figure 1d). The SPAD (soil and plant analyzer development) value of *ltr1* was significantly higher than that of NPB (Figure 1e). The quantum efficiency of photosystem II (Fv/Fm) and leaf water content of *ltr1* were significantly lower than those of NPB, decreased by 8.69% and 5.26%, respectively (Figure 1f,g). These results showed that growth and development of *ltr1* were seriously impaired. The abnormal leaf morphology of *ltr1* was associated with lower light energy conversion efficiency of the PS II (Photosystem II) reaction center and poor leaf water retention.

### 2.2. Effect of LTR1 on Photosynthetic Efficiency and Seed Setting Rate

**Figure 2:**
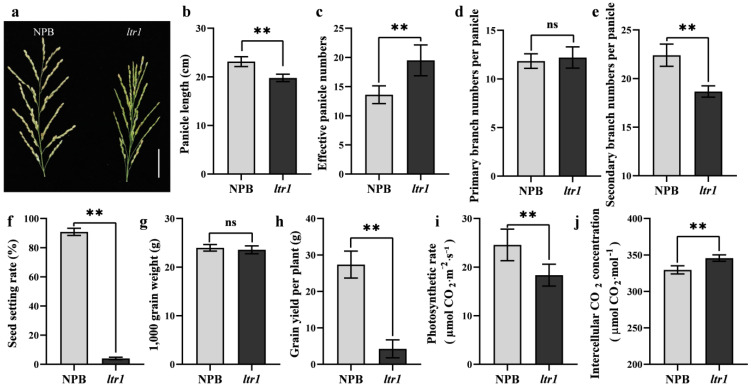
Comparisons of yield characters in NPB and *ltr1*. (**a**) Spike morphology, bar = 4 cm, (**b**) panicle length, (**c**) numbers of effective panicle, (**d**) number of primary branches, (**e**) number of secondary branches, (**f**) seed setting rate, (**g**) 1000-grain weight, (**h**) grain yield per plant, (**i**) photosynthetic efficiency, and (**j**) intercellular CO_2_ concentration of NPB and *ltr1*. Data are given as means ± SD. Asterisks indicate significant difference based on the Student’s *t*-test: ** in the figure represents significant difference at *p* < 0.01 and ns in the figure represents there is no significant different at *p* < 0.05.

According to our results, the panicle length (Figure 2a,b), seed setting rate (Figure 2f), secondary branch numbers (Figure 2e), and grain yield per plant (Figure 2h)were significantly lower in *ltr1* plants than in the wild type, by 14.50%, 95.54%, 16.67%, and 84.49%, respectively. The effective panicle number was significantly higher for *ltr1* than for NPB, with a 43.38% increase (Figure 2c). The primary branch numbers and 1000-grain weight showed no significant differences (Figure 2d,g). These results indicated that the decrease of yield per plant in *ltr1* was caused by the extremely low seed-setting rate and showed that *ltr1* had serious defects in leaf morphology and fertility.

Photosynthesis is the sum of a series of complex metabolic reactions [37]. Maintaining high chlorophyll content in leaves is not necessary to improve the effective photosynthetic rate. Light intensity under low-light conditions is a limiting factor for leaf photosynthesis, and high chlorophyll content is conducive to light absorption; the photosynthetic rate under saturated light intensity is mainly affected by the catalytic ability of the Rubisco enzyme, rather than the limitation of electron transfer rate in light reactions [38]. To explore whether the increased chlorophyll content and abnormal leaf morphology of *ltr1* affect photosynthetic efficiency, we measured the photosynthetic efficiency of NPB and *ltr1* in the field. Compared to NPB, the intercellular CO_2_ concentration of *ltr1* was 4.91% higher, and the photosynthetic efficiency was 25.33% lower (Figure 2i,j). Although the photosynthetic pigment content of *ltr1* increased, the photosynthetic efficiency did not. The reasons for the decrease of photosynthetic efficiency in *ltr1* require further exploration.

### 2.3. Map-Based Cloning of LTR1

**Figure 3:**
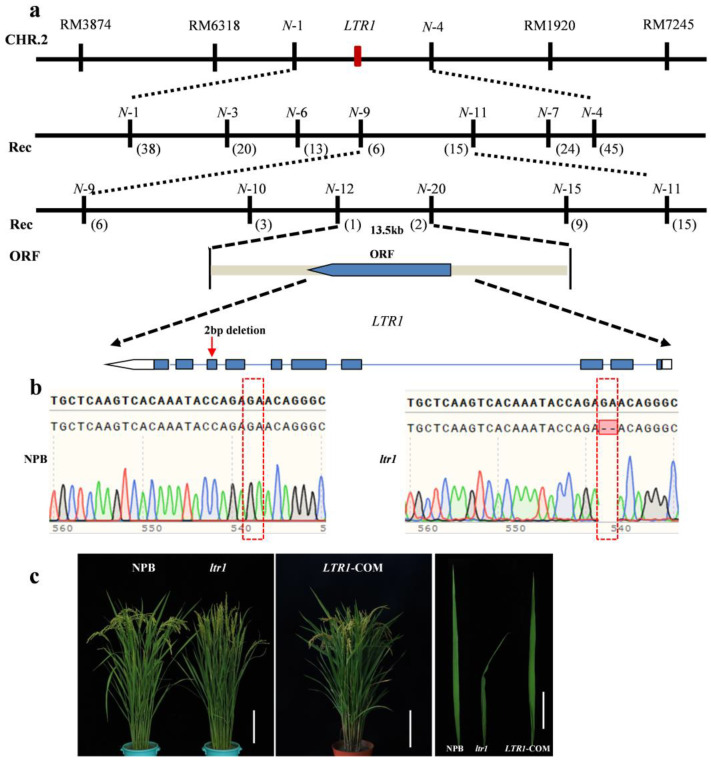
Map-based cloning of *LTR1*. (**a**) Fine mapping of *LTR1*; the red arrow represents the mutation site of *LTR1* in *ltr1*. (**b**) Sequence analysis of NPB and *ltr1*; the red box represents the mutation site in *ltr1*. (**c**) Complementary analysis of *LTR1* in *ltr1*; bar for plants and leaves was 20 cm and 5 cm, respectively.

To explore the molecular mechanism of the phenotype in *ltr1*, an F_2_ segregation population was developed by crossing *ltr1* and the *indica* cultivar TN1. The segregation of wild type and mutant phenotype displayed a ratio of 3:1 (Table S1), indicating that the mutant phenotype was controlled by a single recessive gene. Using 21 F_2_ mutant individuals, the *LTR1* locus was first mapped to the region between RM6318 and RM1920 on the long arm of chromosome 2. The location was then narrowed down to a 13.5-kb genomic region between the markers N-12 and N-20 (Figure 3a). In this region, only one putative opening reading frame (ORF) was found based on data from the Rice Genome Annotation Project (http://rice.plantbiology.msu.edu accessed on 24 March 2021) database. DNA sequence analysis of the ORF in *ltr1* and NPB revealed that a 2-bp deletion in exon 8 of *LOC_Os02g40784*, which resulted in a frameshift mutation and early termination of transcription (Figure 3b). *LOC_Os02g40784* includes ten exons and nine introns and encodes a polypeptide 619 amino acid in length. We therefore inferred that *LOC_Os02g40784* was the gene controlling the mutant phenotype of *ltr1*.

To confirm that the phenotype of *ltr1* was attributable to the detected mutation in *LTR1*, we constructed a complementation vector with a NPB genomic fragment containing the entire coding region of *LTR1* and obtained complementary plants of *LOC_Os02g40784* under *ltr1* background. As expected, the complementary transgenic T_0_ plants showed normal flat leaves: this indicated that the normal expression of *LOC_Os02g40784* in *ltr1* can complement the phenotype of the mutant (Figure 3c).

### 2.4. Overexpression and Targeted Deletion of LTR1

**Figure 4:**
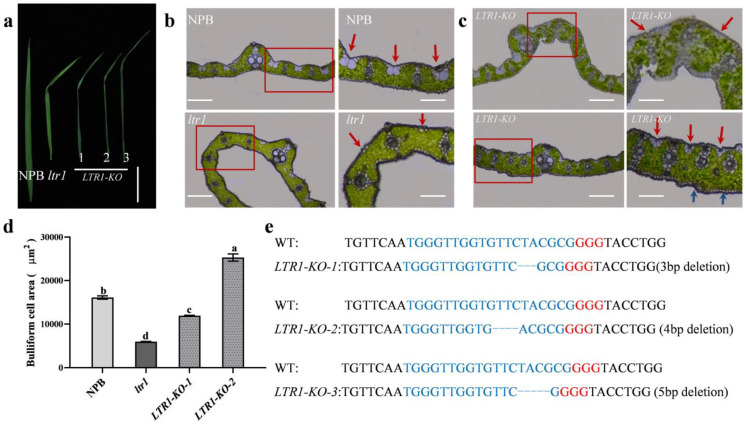
Phenotypic investigation of *LTR1* knockout lines. (**a**) Photos of leaves in NPB, *ltr1*, and *LTR1-KO* lines, bar = 8 cm. (**b**,**c**) Frozen section analysis of leaf in NPB, *ltr1*, and *LTR1-KO* lines; the red arrow represents bulliform cells, and the blue arrow represents the location of sclerenchyma cells. Right of (**b**) is the enlarged detail of red box in the left of (**b**), bar = 200 μm. Right of (**c**) is the enlarged detail of red box in the left of (**c**), bar = 100 μm. (**d**) The area of bulliform cells of *LTR1* knockout lines. (**e**) Sequence analysis of WT and *LTR1-KO*. Data are given as means ± SD. Significant differences were determined by Duncan’s new multiple range test and indicated with different lowercase letters (*p* < 0.05).

**Figure 5:**
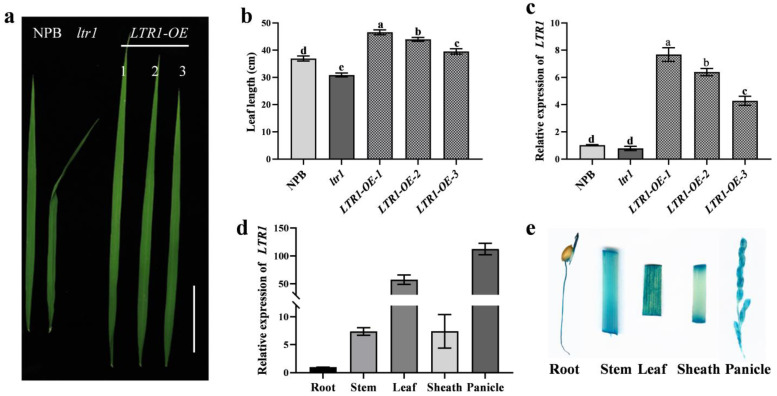
Overexpression and expression pattern analysis of *LTR1*. (**a**) Phenotypic investigation of overexpression lines of *LTR1*, bar = 6 cm. (**b**) Leaf length of overexpression lines. (**c**) The relative expression level of *LTR1* in overexpression lines. (**d**) The relative expression level of *LTR1* in different organs of NPB. (**e**) Promoter activities of *LTR1* in different organs of NPB as determined by promoter–GUS assays. Data are given as means ± SD. Significant differences were determined by Duncan’s new multiple range test and indicated with different lowercase letters (*p* < 0.05).

We next used CRISPR/Cas9 to generate mutant alleles of *LTR1* alleles in a NPB background. We obtained three independent transgenic lines that all carried homozygous mutants, including 3-bp, 4-bp, and 5-bp deletions in exon 3, respectively (Figure 4a,e). These lines had comparable phenotypes to those of *ltr1* with shrunken and distorted leaves, uneven distribution of bulliform cells on adaxial surface and sclerenchyma cells on abaxial surface, and disordered vascular bundles (Figure 4a–d). We also generated overexpression line of *LTR1* in the NPB background, which exhibited longer leaves and higher relative expression level (Figure 5a–c). These results showed that *LOC_Os02g40784* was *LTR1* and that the mutation in *LOC_Os02g40784* led to rumpled leaf phenotype in *ltr1*. Moreover, we found that compared with NPB, the grain yield per plant in overexpression of *LTR1* increased by 38.59% (*p* < 0.05), but the grain yields per plant in *ltr1* and *LTR1-KO* decreased by 82.13% and 76.31% (*p* < 0.05), respectively (Figure S6), suggesting that overexpression of *LTR1* enhanced yield in rice.

To examine the expression pattern of *LTR1* in NPB, total RNA was extracted from roots, stem, leaf, sheath, and panicles. The qRT-PCR showed that *LTR1* was constitutively expressed in all of the tested tissues, with a dramatic increase in leaves and panicles (Figure 5d). The results were consistent with those of β-glucuronidase (GUS) staining (Figure 5e) and the decreased seed-setting rate of *ltr1* (Figure 2f), showing the important regulatory role of *LTR1* in leaf and panicle development.

### 2.5. Phylogenetic Analysis of LTR1

**Figure 6:**
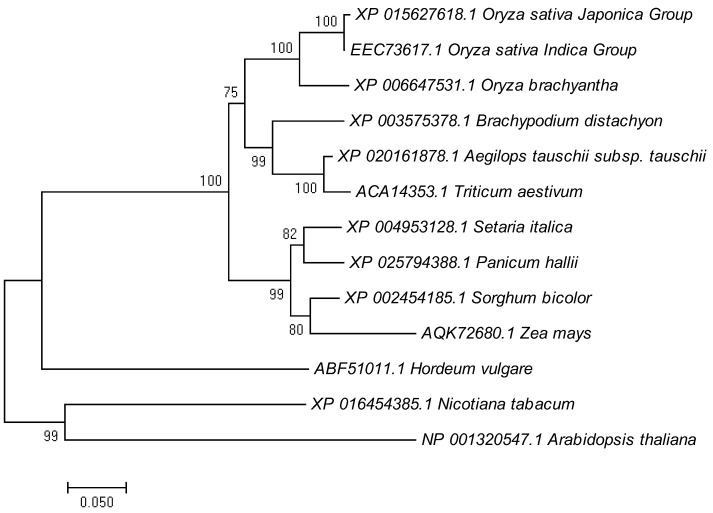
Phylogenic tree of LTR1 and its homologs. The tree was constructed using MEGA 7.0. Protein sequences are *Oryza sativa Japonica* Group (XP 015627618.1), *Oryza sativa Indica* Group (EEC 73617.1), *Oryza brachyantha* (XP 006647531.1), *Brachypodium distachyon* (XP 003575378.1), *Aegilops tauschii* (XP 020161878.1), *Triticum aestivum* (ACA 14353.1), *Setaria italic* (XP 004953128.1), *Panicum hallii* (XP 025794388.1), *Sorghum bicolor* (XP 002454185.1), *Zea mays* (AQK 72680.1), *Hordeum vulgare* (ABF 51011.1), *Nicotiana tabacum* (XP 016454385.1), *Arabidopsis thaliana* (NP 001320547.1). Scale represents percentage substitutions per site. Statistical support for the nodes is indicated.

Protein domain predictions using NCBI CD Search (https://www.ncbi.nlm.nih.gov/Structure/cdd/wrpsb.cgi accessed on 10 August 2018) showed that LTR1 contained ERG3 (elicitor-responsive genes, ERG) and wax2_C domains. BLAST-P analysis of the NCBI database showed that LTR1 was highly conserved in higher plants including *Oryza brachyantha* (92.25%), *Brachypodium distachyon* (84.98%), *Aegilops tauschii* (84.87%), *Triticum aestivum* (83.84%), *Setaria italic* (82.90%*), Panicum hallii* (81.42%*), Sorghum bicolor* (82.23%), and *Zea mays* (78.33%) (Figure S1). To investigate the evolutionary relationships between LTR1 homologs, a phylogenic analysis was performed using the Text Neighbor-Joining Tree method [39]. The results showed that LTR1 is closely related to homologues in the grass family containing *Aegilops tauschii*, *Brachypodium distachyon*, and *Triticum aestivum* (Figure 6 and Figure S1). Overall, these analyses demonstrated that the LTR1 was highly conserved in plants.

### 2.6. LTR1 Participates in Water Transport and Ion Homeostasis

RNA-seq analysis showed that there were 6513 differentially expressed genes (DEGs) in NPB and *ltr1*, of which 3022 were up-regulated and 3480 were down-regulated (Figure S2a and Table S6). There were 118 DEGs related to leaf development, comprising 36 up-regulated and 82 down-regulated genes (Figure S3a and Table S7). A Kyoto Encyclopedia of Genes and Genomes (KEGG) pathway analysis showed that these DEGs were mainly enriched in plant hormone signal transduction pathways, which indicated that *LTR1* may regulate leaf development by participating in hormone signal transduction pathways (Figure S3b). For example, BR C-6 oxidase gene (*OsBR6ox*), *AUXIN RESPONSE FACTOR8* (*OsARF8*), *AUXIN RESPONSE FACTOR17 (OsARF17*), *AUXIN RESPONSE FACTOR16 (OsARF16*), *PHYTOSULFOKINE RECEPTOR 2* (*OsPSKR2*), and *PHYTOSULFOKINE RECEPTOR 3 (OsPSKR3*) were up-regulated (Figure S3c) and *PENTATRICOPEPTIDE REPEAT PROTEIN* (*OsPPR6*), *RNA-dependent RNA polymerase 6* (*OsRDR6*), *RNA-directed RNA polymerase 1* (*OsRDR1*), *INCREASED LEAF ANGLE1* (*ILA1*), *dwarf 11* (*d11*), and *GIBBERELLIN 20-OXIDASE GENE* (*OsGA20ox1*) were down-regulated in *ltr1* plants (Figure S3d).

**Figure 7:**
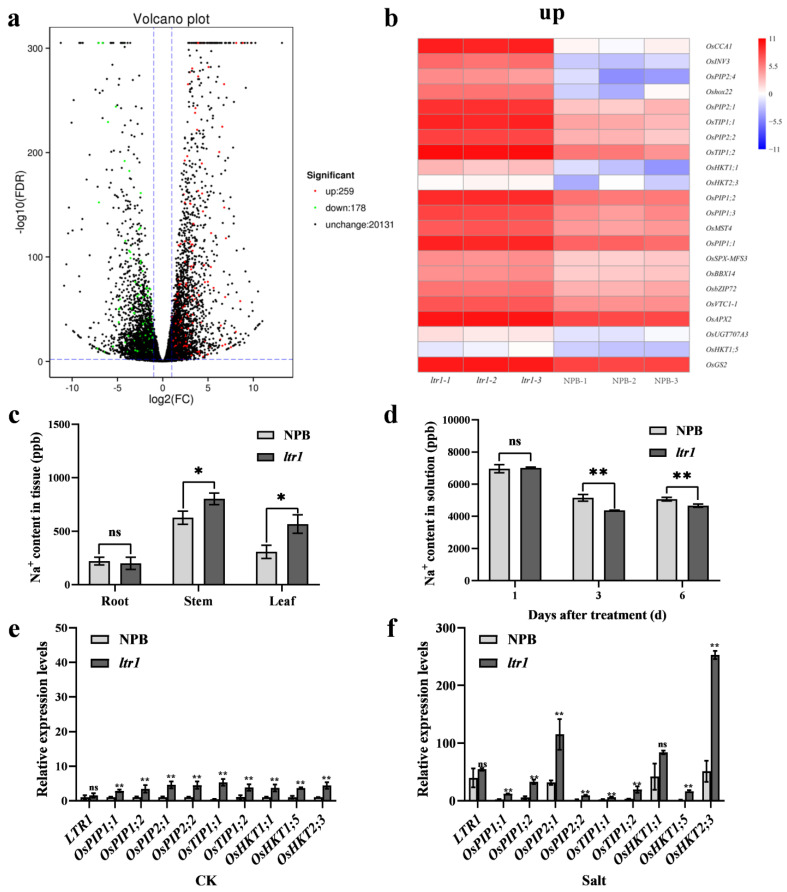
*LTR1* regulates salt-stress response by regulating genes encoding aquaporins and ion transporters. (**a**) Volcano plot of DEGs related to salt response between NPB and *ltr1*. (**b**) Heat map of significantly up-regulated DEGs encoding aquaporin and ion transporters between NPB and *ltr1*. (**c**) Na^+^ content in different tissues of NPB and *ltr1*. (**d**) Na^+^ content in solutions where NPB and *ltr1* were cultured after treatment for 1, 3, or 6 d. (**e**) Relative expression levels of *LTR1* and genes encoding aquaporin and ion transporters under normal condition (CK). (**f**) The relative expression levels of *LTR1* and genes encoding aquaporin and ion transporters under 150 mM NaCl (Salt). Data are given as means ± SD. Asterisks indicate significant difference based on the Student’s *t*-test: * in the figure represents significant difference at *p* < 0.05; ** in the figure represents significant difference at *p* < 0.01 and ns in the figure represents there is no significant difference at *p* < 0.05.

A Gene Ontology (GO) term enrichment analysis was also conducted for DEGs between NPB and *ltr1*. The most highly enriched GO biological processes were in salt-stress response, stimulus response, and ABA response (Figure S2b). The most highly enriched GO molecular functions were ATP binding and protein binding, and the most enriched cell components were plasma membrane and nucleus (Figure S2c,d). These results suggested that *LTR1* was involved in the salt-stress response. It was previously reported that plant membrane transporters play key roles in resistance to biological and abiotic stresses; in particular, Na^+^/K^+^ transporters increase resistance to salt stress [40]. We further found that there were 259 up-regulated and 178 down-regulated DEGs related to the salt-stress response (Figure 7a and Table S8). In *ltr1*, most of the genes encoding aquaporin or related to Na^+^/K^+^ transporters were up-regulated, such as *PLASMA MEMBRANE INTRINSIC PROTEIN genes OsPIP1;1*, *OsPIP1;2*, *OsPIP1;3*, *OsPIP2;1*, *OsPIP2;2*, *OsPIP2;4*, *OsPIP2;4*; *TONOPLAST INTRINSIC PROTEIN* genes *OsTIP1;1*, *OsTIP1;2*; *HIGH-AFFINITY K^+^ TRANSPORTERS* genes *OsHKT1;14*, *OsHKT2;3*, and *OsHKT1;5* (Figure 7b). These results suggested that *LTR1* may affect salt tolerance by regulating water transport and ion homeostasis in plants through aquaporin and Na^+^/K^+^ transporters. Given that many genes encoding aquaporins and ion transporter were differentially expressed in NPB and *ltr1*, we considered the possibility that *LTR1* may regulate salt tolerance by affecting water transport and ion homeostasis. Therefore, we measured the Na^+^ content in solution and in tissues of NPB and *ltr1* under salt stress. After salt stress, Na^+^ content in stems and leaves of *ltr1* were significantly higher than those of NPB, which increased by 28.24% and 45.75%, respectively (*p* < 0.05). There was no significant difference in Na^+^ content in the roots of NPB and *ltr1* (*p*< 0.05) (Figure 7c). Furthermore, there was no significant difference in Na^+^ content in the liquid media in which NPB and *ltr1* plants were grown after treatment in hydroponic solution for 1 d (*p* < 0.05). However, after treatment for 3 or 6 d, Na^+^ content was lower in the solution in which *ltr1* plants were grown compared to NPB plants, decreased by 15.20% and 8.03%, respectively (*p* < 0.01) (Figure 7d). Under normal growth conditions (CK), the relative expression levels of *OsPIP1;1*, *OsPIP1;2*, *OsPIP2;1*, *OsPIP2;2*, *OsTIP1;1, OsTIP1;2*, *OsHKT1;1*, *OsHKT1;5*, and *OsHKT2;3* were significantly higher in *ltr1* than NPB leaves (Figure 7e), increased by 1.85, 2.36, 3.48, 3.46, 10.2, 2.57, 2.67, 2.52, 3.44 times, respectively (*p* < 0.01); which was consistent with the RNA-seq results. The genes encoding aquaporin and ion transporter in NPB and *ltr1* plants both were strongly induced by salt stress. However, the induction of these genes was stronger in *ltr1* than in leaves of NPB, leading to relative expression levels of *OsPIP1;1*, *OsPIP1;2*, *OsPIP2;1*, *OsPIP2;2*, *OsTIP1;1*, *OsTIP1;2*, *OsHKT1;5*, and *OsHKT2;3* that were significantly higher in *ltr1* than NPB leaves under salt stress (*p* < 0.01), especially the expression of *OsPIP2;1* and *OsHKT2;3* (Figure 7f). This was consistent with the finding that the Na^+^ content in stems and leaves of *ltr1* were significantly higher than those of NPB.

### 2.7. LTR1 Regulates Salt Tolerance in Rice

**Figure 8:**
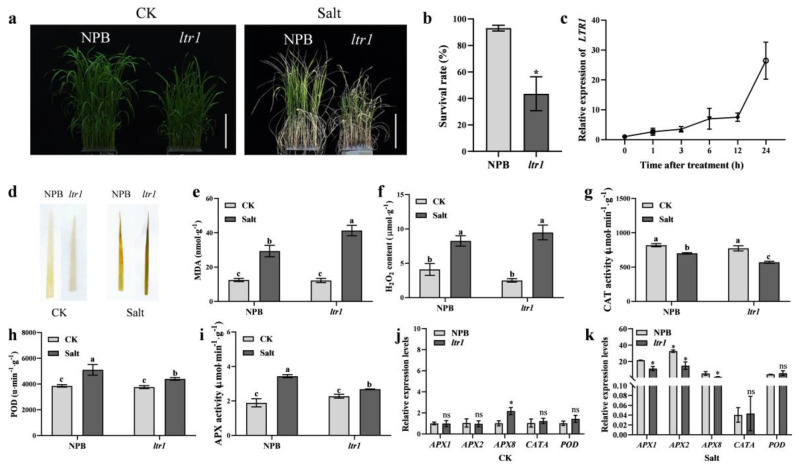
The response of *LTR1* to salt stress in NPB and *ltr1*. (**a**) Photos of NPB and *ltr1* under CK and Salt treatment, bar = 10.5 cm. (**b**) The survival rate of NPB and *ltr1* after treatment for 7 d. (**c**) The relative expression level of *LTR1* after treatment for 0, 1, 3, 6, 12, 24 h. (**d**) DAB staining in leaves of NPB and *ltr1* under CK and salt treatment. (**e**,**f**) MDA and H_2_O_2_ content in leaves of NPB and *ltr1* under CK and Salt treatment. (**g**–**i**) CAT, POD, and APX activity in leaves of NPB and *ltr1* under CK and salt treatment. (**j**,**k**) The relative expression level of genes related to antioxidant system in leaves of NPB and *ltr1* under CK and Salt treatment, *n* = 4. Data are given as means ± SD. Asterisks indicate significant difference based on the Student’s *t*-test: * in the figure represents significant difference at *p* < 0.05 and ns in the figure represents there is no significant difference at *p* < 0.05. Different lowercase letters indicate significant differences based on the Duncan’s new multiple range test (*p* < 0.05).

To further explore the function of *LTR1* in the salt-stress response, we first screened a suitable salt concentration for treatment. NPB and *ltr1* were cultured in soil treated with 0 mM NaCl (CK treatment), 50 mM NaCl, 100 mM NaCl, or 150 mM NaCl at the five-leaf stage. Two weeks later, the survival rates of NPB treated with 150 mM NaCl was higher than that of *ltr1* plants (92.30% and 64.30%, respectively) (*p* < 0.05) (Figure S4). We then grew NPB plants in solution, treated them with 150 mM NaCl, and measured the relative expression level of *LTR1* at 0, 1, 3, 6, 12, and 24 h. The relative expression level of *LTR1* increased overtime; the relative expression level of *LTR1* increased by 6.95 times at 6 h and by 26.29 times at 24 h after treatment, indicating that *LTR1* was significantly induced by salt stress (Figure 8c). After 7 d of salt stress in hydroponic solution, the survival rate of NPB reached 93.05%, which was significantly higher than that of *ltr1* (43.52%) (*p* < 0.05) (Figure 8b). After 3 d of salt stress in hydroponic solution, H_2_O_2_ and MDA in levels of NPB and *ltr1* both accumulated, and the accumulation of MDA in the leaves of *ltr1* was significantly higher than that of NPB (*p* < 0.05) (Figure 8d–f). These results suggested that, compared with NPB, the membrane lipid peroxidation and plasma membrane damage in *ltr1* were more serious after salt stress, and that *ltr1* was more sensitive to salt stress (Figure 8a–f). Studies have shown that when plants are subjected to stress, the enzymatic protection system is initiated rapidly, and the activities of peroxidase (POD), ascorbate peroxidase (APX), and other enzymes increase significantly, which enhances the capacity for reactive oxygen species (ROS) scavenging and reduces damage [41,42,43]. In this study, after salt stress, the catalase (CAT) activity in NPB and *ltr1* decreased by 14.38% and 26.17%, respectively (*p* < 0.05). The decrease of CAT activity in *ltr1* was more significant (Figure 8g). Furthermore, the activities of POD and APX in NPB and *ltr1* both increased after stress, and the increases in POD and APX activities induced by stress in *ltr1*were weaker than that in NPB. After salt stress, the POD and APX activities of NPB increased by 32.31% and 81.62% compared with CK, while the POD and APX activities in *ltr1* increased by 16.97% and 18.01% (*p* < 0.05) (Figure 8h,i). These were consistent with the expression change of antioxidant system in leaves of NPB and *ltr1* (Figure 8j,k). Therefore, these results indicated that *ltr1* had an inferior ability to adapt to salt stress.

## 3. Discussion

### 3.1. LTR1 Encodes a Wax Synthesis Gene and Regulates Leaf Morphology

Cell structure is a key factor regulating leaf morphology. Many cloned genes regulated leaf morphology through affecting the normal development of vascular bundles, sclerenchyma cells, bulliform cells, epidermis, and cell walls [9]. However, few of these genes that affect leaf shape are involved in wax synthesis. In this study, we cloned a leaf shape gene, *LEAF TIP RUMPLED1* (*LTR1*), which is an allele of the wax synthesis gene *OsGL1-4* [44]. *LTR1* regulated leaf morphology, and loss function of *LTR1* led to rumpled leaves with the abnormal development of bulliform cells, vascular bundles, and sclerenchyma cell. These indicated that *LTR1* affected leaf morphology by regulating the development of bulliform cells, vascular bundles, and sclerenchyma cell. BR signal and auxin metabolism pathway played important roles in leaf morphogenesis [8,45]. *OsBR6ox*, which participates in brassinosteroid (BR) biosynthesis and signal transduction pathway, regulated normal development of organs and then induced abnormal vascular tissue and twisted leaves in its loss-of-function mutant [46]. *OsARF16* [47] and *OsARF17* [48] participate in the auxin response, affecting auxin polar transport and vascular tissue development. The RNA-dependent RNA polymerase OsRDR6 participates in formation of trans-acting small interfering RNA (ta-siRNA) [49], and ta-siRNA inhibits ARF3/ARF4 expression and thus inhibits maintenance of abaxial polarity [50]. *OsAGO7*, a *ZIP*/*Ago7* homolog in *Arabidopsis thaliana*, is a critical member of the ta-siRNA-ARF3/ARF4-OsAGO7 complex and participates in regulation of leaf rolling [51]. In this study, *OsBR6ox*, *OsARF16*, *OsARF17* and *OsRDR6* were found to be differentially expressed in NPB and *ltr1*. We therefore speculated that *LTR1* may affect leaf morphology by participating in plant hormone signal transduction pathway, while the detailed regulatory network involved requires further study.

### 3.2. LTR1 Has Multiple Effects on Plant Growth and Development

There are 11 Glossy1 (*GL1*) homologous genes in rice, *OsGL1-1* through *OsGL1-11*, which vary expression levels between rice tissues and organs. Most are induced by abiotic stress and play key roles in wax synthesis and stress tolerance [44]. It was reported that *OsGL1-1*, *OsGL1-2*, *OsGL1-3*, and *OsGL1-6* affect the leaf water loss rate by controlling the wax content in the leaf epidermis, thereby controlling drought resistance in rice [31,44,52,53]. In the present study, we found that *LTR1*, an allele of *OsGL1-4*, was also involved in the regulation of salt tolerance with *LTR1* strongly induced by salt stress. The *ltr1* plants showed high sensitivity to salt stress compared to the wild-type, with more serious membrane lipid peroxidation and plasma membrane damage. Moreover, in rice, many humidity-sensitive genic male sterile lines (HGMS) were obtained by identifying wax synthesis genes involved in regulating pollen development and affecting panicle fertility. Previous studies have shown that most wax synthesis genes, such as *DROUGHT HYPERSENSITIVE* (*DPS1*) [32], *SUBTILISIN-LIKE SERINE PROTEASE 1 (SUBSrP1)* [54], *HMS1-INTERACTING PROTEIN (HMS1I)* [55], *HUMIDITY-SENSITIVE GENIC MALE STERILITY 1 (HMS1)* [56], and *OsGL1-5* [44] were involved in the regulation of panicle fertility. Loss functions of these genes resulted in abnormal pollen development and a decrease in the seed setting rate at low humidity but a normal seed setting rate at high humidity. Based on this mechanism, the corresponding mutants can be used as HGMSs. It has also been reported that *OsGL1-4* controls male sterility in rice by affecting pollen adhesion and water cooperation under ambient humidity [57]. We here found that loss function of *LTR1* resulted in a severe decrease in the seed setting rate and grain yield per plant, and significant changes in the number of branches and effective panicles in *ltr1*. What’s more, overexpression of *LTR1* enhances yield in rice. These results indicated that *LTR1* had pleiotropic functions in rice growth and development.

### 3.3. LTR1 Regulated Salt Tolerance by Altering Plant Water Status and Ion Homeostasis

Plant aquaporins play very important roles in water transport of transmembrane and form a large protein family [58]. Great progress has been made in functional studies of plasma membrane intrinsic proteins (PIPs) and tonoplast intrinsic proteins (TIPs), which have shown that their main physiological function is to promote transmembrane transport of osmotic water [58]. The expression regulation of *PIPs* varies with differing experimental conditions [59]. *OsPIP1;1* showed low water channel activity in *Xenopus oocytes*, but the permeability of OsPIP1;1 improved significantly when it was co-expressed with *OsPIP2.1* [60]. In the present study, the relative expression level of *OsPIP2;1* was much higher than that of *OsPIP1;1, OsPIP1;2*, and *OsPIP2;2*). This indicated that the upregulation of *OsPIP2;1* resulted in enhanced leaf permeability and poor water retention in *ltr1*. Class I HKT transporters play an important role in removing sodium ions from the xylem [61,62]. Because the accumulation of K^+^ in plant cells homeostasis the toxicity of Na^+^ accumulation, stable acquisition and distribution of K^+^ are required during salt-stress conditions [63]. The OsHKT transporter is involved in Na^+^ transport in rice, and OsHKT1 specifically mediates Na^+^ uptake by rice roots under conditions of K^+^ deficiency [64]. *OsHKT1;5* controls the transport of K^+^ and Na^+^ from roots to shoots. Under salt stress, *OsHKT1;5* refluxes of excessive Na^+^ from shoots to roots by unloading it from the xylem, thereby reducing Na^+^ toxicity and enhancing salt tolerance [61]. However, we here found that high expression of *OsHKT1;5* under high salt conditions did not reduce the accumulation of Na^+^ in *ltr1* leaves. Thus, the excessive accumulation of Na^+^ in *ltr1* under salt stress may be regulated by other factors. Under salt stress, the relative expression of *HKT2;3* was significantly higher than the expression of other genes encoding ion transporters. Meanwhile, overexpression of the aquaporin gene *OsPIP2;1* led to enhanced water permeability and poor water retention in *ltr1*. More Na^+^ was absorbed by *ltr1* than NPB roots and transported to aboveground parts; thus, the Na^+^ content was significantly higher in stems and leaves of *ltr1* than NPB. Furthermore, there were more white crystals on the stems of NPB than that of *ltr1* (Figure S5). These results suggested that over-accumulation of Na^+^ in *ltr1* could not be reversed in a timely fashion, resulting in high sensitivity of *ltr1* to salt stress. Therefore, we speculated that *LTR1* affected the water status and ion homeostasis of plants by regulating the expression of genes encoding aquaporins and ion transporters, which ultimately regulated salt tolerance in plants.

### 3.4. Prospects

Wax, cuticle, and polysaccharide form the cuticle of epidermis, which is a self-protective barrier against biotic and abiotic stresses in plants [29,65,66]. Wax affects canopy temperature and water transport in plants, which further affect plants adaptation to harmful environmental factors such as heat/drought/salt stress and pest/pathogen damage [29,32]. Here, we found that the wax synthesis gene *LTR1* regulates leaf morphology by affecting the normal development of bulliform cells, vascular bundles, and sclerenchyma cells. Moreover, overexpression of *LTR1* enhanced yield in rice and *LTR1* positively regulates salt stress by affecting water and ion homeostasis in plants. However, the regulatory and response mechanism by which *LTR1* affected leaf morphogenesis, water retention, and ion transport between the root and shoot requires further analysis. The differences in ion transport (ion flow rate, ion transport efficiency) and horizontal balance ability between NPB and *ltr1*, together with their regulatory mechanisms need to be further analyzed. How wax content affects cell structure, tissue moisture, and ion balance need further exploration. Identifying proteins that directly interact with LTR1 and analyzing the molecular mechanism of their interaction in regulating leaf shape and salt tolerance will further supplement the known genetic regulation network that governs leaf shape and salt tolerance, providing a theoretical foundation for breeding high-yield rice varieties with high salt tolerance. In addition, identification and application of favorable alleles of *LTR1*, which confers resistances without negative effects on yield, can potentially be used to breed high-yield and high-resistance rice varieties through the combination of multi-omics and bioinformatics. Therefore, according to the insights uncovered in this study, *LTR1* can be considered as a potentially highly valuable gene resource for the improvement of leaf morphology and stress resistance in rice breeding. Manipulating genes associated with leaf morphology and stress resistance individually or in combination makes it possible in the “precision breeding” to breed rice varieties with ideal plant architecture and high resistances without yield penalties. Thus, our results illustrate innovative approaches for developing potentially high stress resistant crop varieties with ideal plant architecture and carry significant implications for breeding application of high yield and stress-resistance-related genetic resources.

## 4. Materials and Methods

### 4.1. Plant Materials and Growth Conditions

In this study, the *ltr1* mutant was isolated from a population of the *Oryza sativa* ssp. *japonica* variety Nipponbare (NPB) mutagenized with a 1% ethyl methanesulfonate (EMS) solution using a forward genetic screen for altered leaf shape. Rice plants were grown under natural environmental conditions in an experimental field at the China National Rice Research Institute in Fuyang District (Zhejiang province, China) and Lingshui (Hainan province, China).

Seedlings used in salt treatments were cultured in soil and hydroponic solution (1.25 mM NH_4_NO_3_, 0.3 mM KH_2_PO_4_, 0.35 mM K_2_SO_4_, 1 mM CaCl_2_, 1 mM MgSO_4_, 0.5 mM NaSiO_3_⸱9H_2_O, 20 μM Fe-EDTA, 9 μM MnCl_2_⸱4H_2_O, 0.39 μM (NH_4_)_6_Mo_7_O_24_⸱4H_2_O, 20 μM H_3_BO_3_, 0.77 μM ZnSO_4_⸱7H_2_O, 0.32 μM CuSO_4_⸱5H_2_O) in an artificial incubator with a 12 h/12 h light/dark at 70–80% humidity and a 25–30 °C/28 °C day/night temperature (MLR-352H-PC, Panasonic, Osaka, Japan). For salt-stress treatments, plants were cultivated in hydroponic media containing 0 mM NaCl (CK), 50 mM NaCl, 100 mM NaCl, or 150 mM NaCl (Salt), respectively.

### 4.2. Phenotypicl Characterization and Histological Analysis

To investigate whether the *LTR1* mutation affected rice yield, agronomic traits such as panicle length, effective panicle number, numbers of branches, grain numbers per panicle, seed setting rate, grain yield per plant, and 1000-grain weight were measured for each of 5 or 6 biological replicates at the mature stage. The panicle length, number of branches, and grain numbers per panicle were obtained from measurements of the main panicle. 

For frozen cross-section assays, the leaves were immersed in frozen embedding agent (Tissue-Tek^®^ O.C.T. Compound, Sakura, Tokyo, Japan) for 2–3 h at –20 °C. Sections (15 μm) were cut with a freezing microtome (Leica CM1950, Wetzlar, Germany) and placed on microscope slides. Slices were observed and photographed using a microscope (Leica DM4 B). The areas of bulliform cells were calculated using Image J software.

### 4.3. Measurements of Chlorophyll Content and Photosynthetic Parameters

Chlorophyll *a*, Chlorophyll *b*, and carotenoid (Car) content were measured in three biological replicates using the methods described by Sartory and Grobbelaar [67].

SPAD values were determined for ten biological replicates using a SPAD-502 PLUS. Chlorophyll fluorescence was measured for ten biological replicates with a FluorPen FP100. The QY (Fv/Fm) was determined after a 20 min dark adaptation period.

The net photosynthesis rate, stomatal conductance, and transpiration rate of NPB and *ltr1* plants were evaluated for eight biological replicates with a Li-COR 6400 portable system. All measurements were conducted under the following conditions: photosynthetic photon flux density of 1200 μmol⸱m^−2^⸱s^−1^, ambient CO_2_ (400 μmol⸱mol^−1^), 6 cm^2^ of leaf area, 500 μmol⸱s^−1^ flow speed, and ambient temperature.

### 4.4. Map-Based Cloning and Complementation Assay

To fine-map the mutated gene, an F_2_ population was constructed from a cross between *ltr1* and a wild-type *indica* variety, TN1, with flat leaves. Plants from this population that exhibited rumpled leaves were selected for gene mapping. The locus was first mapped to an interval between the two markers RM6318 and RM1920 (Table S2) on the long arm of chromosome 2, then was further narrowed down to a 13.5-kb DNA region. There was only one open reading frame (ORF) in this region. Genomic DNA fragments in this region were amplified using primers listed in Table S3 from NPB and *ltr1*.

An 8628-bp genomic DNA fragment containing the coding region of *LOC_Os02g40784*, plus 2060-bp upstream, 5450-bp of the coding region and 1118-bp downstream regions, was amplified from NPB (primers for this process are shown in Table S4) and then was cloned into the binary vector pCAMBIA1300 by homologous recombination. The resulting construct pCAMBIA1300-LTR1 was transformed into *ltr1* calli to obtain complementary transgenic plants.

### 4.5. Gene Editing and Overexpression

For generation of knockout plants using CRISPR/Cas9 technology, gene-specific guide sequences (primers are listed in Table S4) targeting *LTR1* were designed to create single guide RNAs (sgRNAs), after which the sgRNA–Cas9 sequences were cloned into pYLCRISPR/Cas9-MH [68].

Full-length cDNA of *LTR1* amplified (primers are listed in Table S4) from NPB was cloned into the Gateway entry vector pDONR ZEO (Invitrogen, Carlsbad, CA, USA), then recombined into the pUbi::attR-GFP-3×FLAG vector using the Gateway cloning system (Invitrogen). The resulting construct was transformed into NPB calli to obtain overexpression lines of *LTR1*.

### 4.6. Histological GUS Assay

The promoter of *LTR1* (2094-bp upstream of the start codon) was amplified from NPB genomic DNA (primers are listed in Table S4) and inserted into the *Eco*RⅠ and *Nco*Ⅰ sites of the binary vector pCAMBIA1305.1. This resulted in a fusion of the promoter and the GUS reporter gene (*pLTR1::GUS*). The recombinant vector was then introduced into NPB calli to obtain transgenic plants.

For GUS staining, different tissues of transgenic plants were incubated in X-Gluc buffer (0.1 mol⸱L^−1^ K_2_HPO_4_ (pH 7.0), 0.1 mol⸱L^−1^ KH_2_PO_4_ (pH 7.0), 5 mmol⸱L^−1^ K_3_Fe (CN)_6_, 5 mmol⸱L^−1^ K_4_Fe (CN)_6_⸱3H_2_O, 0.1% Triton X-100, 20% methanol, and 1 mg⸱mL^−1^ X-Gluc) at 37 °C for 2 h [69]. Stained samples were cleared of chlorophyll by dehydration with ethanol, then scanned using a Microtek Scan Maker i800 plus.

### 4.7. RNA-seq and Data Analysis

Plants were harvested for total RNA extraction at the booting stage. Three biological replicates were used for RNA-seq analysis. The RNA-seq libraries were constructed and sequenced using an Illumine HiSeq. Each sample obtained approximately 20,000,000 clean reads, which were mapped to NPB reference genome based on the genome information by HISAT2 (http://ccb.jhu.edu/software/hisat2/index.shtml accessed on 10 July 2022). Differential expression analysis for NPB and *ltr1* was performed with DESeq2 using thresholds of FDR < 0.01 and |log_2_ (fold change)| ≥ 2). A GO enrichment analysis was implemented with the GOseq R packages. The KEGG pathway analysis of DEGs was conducted using the KEGG database (http://www.genome.jp/kegg/ accessed on 24 March 2021).

### 4.8. Determination of Stress-Related Physiological Index

For 3,3′-diaminobenzidine (DAB) staining, 0.1 g DAB was fully dissolved in ddH_2_O by adjusting pH to 5.8. The samples were immersed into a tube containing 1 mg/mL DAB solution overnight at 28 °C under dark conditions. Stained samples were cleared of chlorophyll by dehydration with 80% ethanol, then scanned using a Microtek Scan Maker i800 plus. The contents of hydrogen peroxide (H_2_O_2_) and malondialdehyde (MDA), activities of catalase (CAT), peroxidase (POD), and ascorbate peroxidase (APX) were measured using appropriate kits from Geruisi (http://www.geruisi-bio.com/ accessed on 18 May 2021) following the manufacturer’s instructions with four biological replicates per sample.

### 4.9. Measurement of Na^+^ Content

For each sample, a total of 0.05 g of dry tissues power were weighed and immersed in 4 mL concentrated nitric acid with 2 mL 30% H_2_O_2_ overnight, and then were decocted with temperature gradient (60 °C for 1 h, 120 °C for 1 h, 160 °C for 1 h, 190 °C until the solution was clarified) using a graphite digestion instrument (DigiBlock ED54, Beijing, China). The content of extracted Na^+^ was measured by inductively coupled plasma-mass spectrometry (ICP-MS) (iCAP RQ, Thermo Fisher Scientific, 168 Third Avenue, Waltham, MA, USA) after acid catching, constant volume and filtration with three biological replicates per sample.

### 4.10. RNA Extraction and Quantitative Real-Time PCR

Total RNA was extracted using the Total RNA Miniprep kit (Axygen, Hangzhou, China) following the manufacturer’s instructions. First-strand cDNA was synthesized using the ReverTra Ace qPCR-RT kit (Toyobo, Osaka, Japan) as instructed by the manufacturer, using 2 μg of total RNA for each reaction. qRT-PCR analyses were performed using SYBR Premix Ex Taq (Takara, Kusatsu, Japan) and gene-specific primers on a CFX96TM real-time system. Three or four biological replicates were performed for all experiments. The primers used are listed in Table S5 in the Supplementary Materials.

### 4.11. Quantification and Statistical Analysis

Quantification analyses on all the measurements were conducted in GraphPad Prism 8. Significant differences were determined with Student’s *t*-test and Duncan’s new multiple range tests.

## Data Availability

Not applicable.

## Supplementary Materials

The following supporting information can be downloaded at: https://www.mdpi.com/article/10.3390/ijms23158818/s1.
