# Supplementary material for: LEAF TIP RUMPLED 1 Regulates Leaf Morphology and Salt Tolerance in Rice
Source: Int J Mol Sci. 2022 Aug 8;23(15):8818. doi: 10.3390/ijms23158818 (PMC9369171; doi:10.3390/ijms23158818)
Supplement: Supplementary file 1 [file ijms-23-08818-s001.zip › Supplementary figures.pdf]

Supplementary Figures

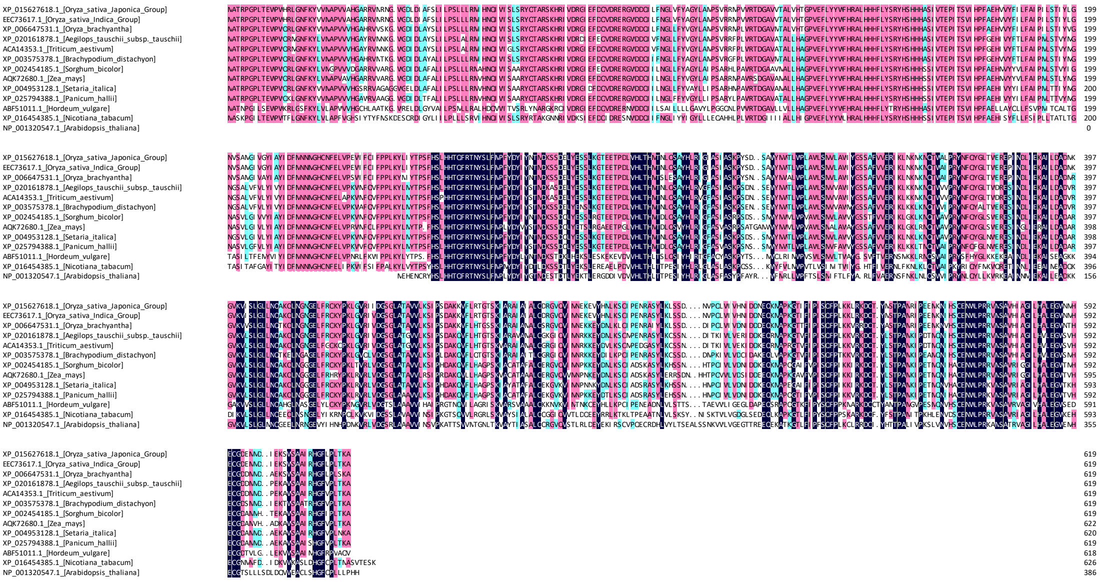

Figure S1. Amino acid sequence alignment of the 12 types of LTR1 homologs. Amino acids that were fully or partially conserved are shaded blue and green, respectively.

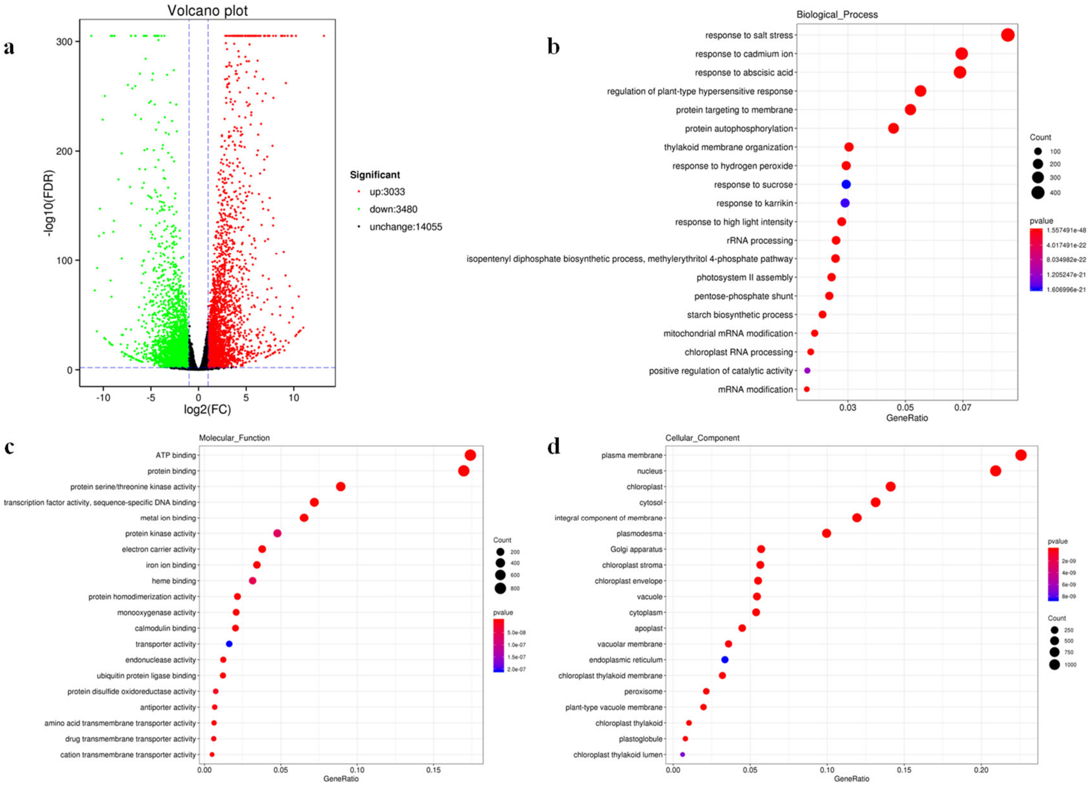

Figure S2. Differentially expressed genes in NPB and *ltr1* by RNA-seq. (**a**) Volcano plot of DEGs between NPB and *ltr1*. (**b**) Bubble diagram for GO enrichment analysis of DEGs in biological processes. (**c**) Bubble diagram for GO enrichment analysis of DEGs in molecular function. (**d**) Bubble diagram for GO enrichment analysis of DEGs in cell component.

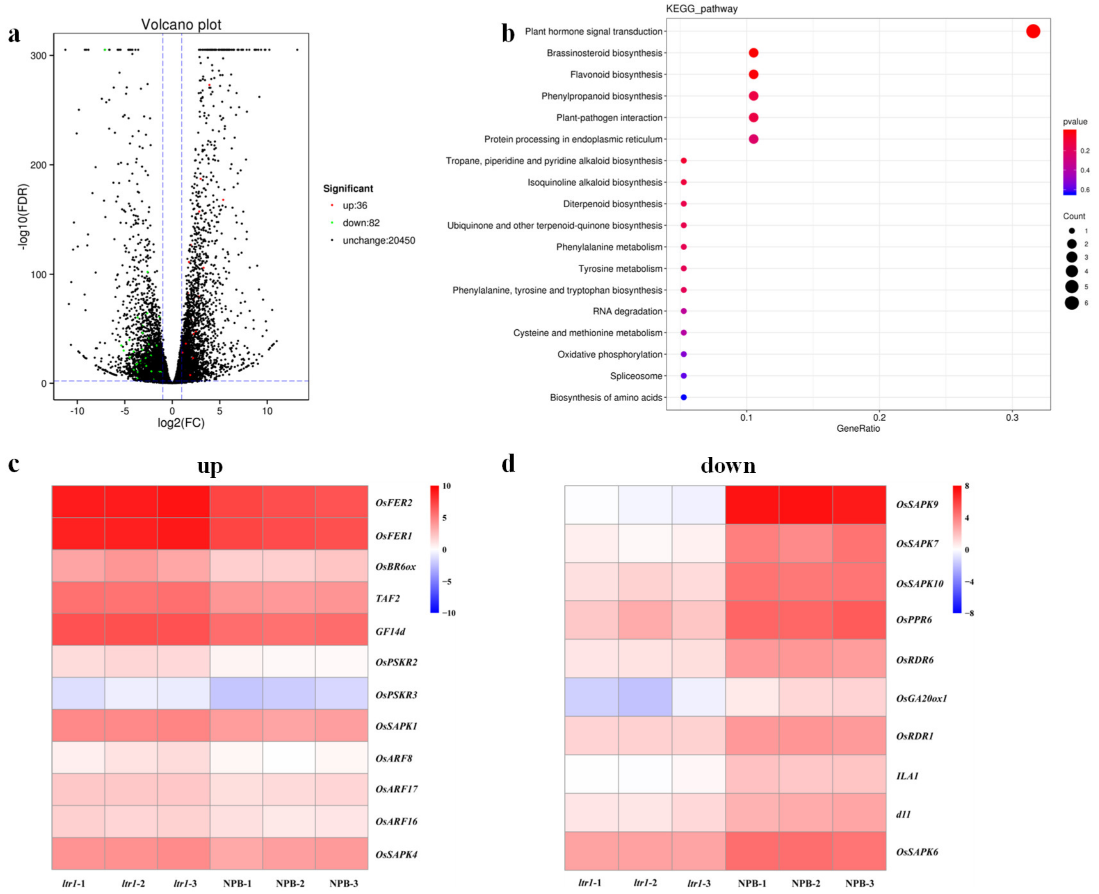

Figure S3. Differentially expressed genes related to leaf development in NPB and *ltr1*. (**a**) Volcano plot of DEGs related to leaf development between NPB and *ltr1*. (**b**) The KEGG pathway analysis of DEGs related to leaf development between NPB and *ltr1*. (**c** and **d**) Heat map of significantly up-regulated and down-regulated DEGs related to leaf development between NPB and *ltr1*.

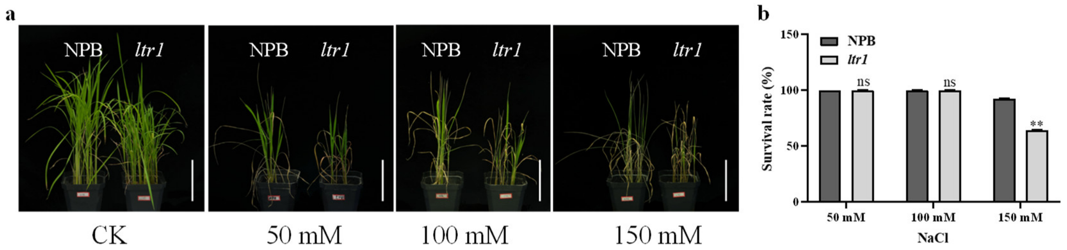

Figure S4. The effects of different concentration of salt on NPB and *ltr1*. (**a**) Plant Morphology of NPB and *ltr1* under different concentration of salt treatment, bar=10 cm. (**b**) The survival rate of NPB and *ltr1* under different concentration of salt treatment. Data are given as means ± SD. Asterisks indicate significant difference based on the Student's *t* - test: ** in the figure represents significant difference at $p < 0.01$ and ns in the figure represents there is no significant difference at $p < 0.05$.

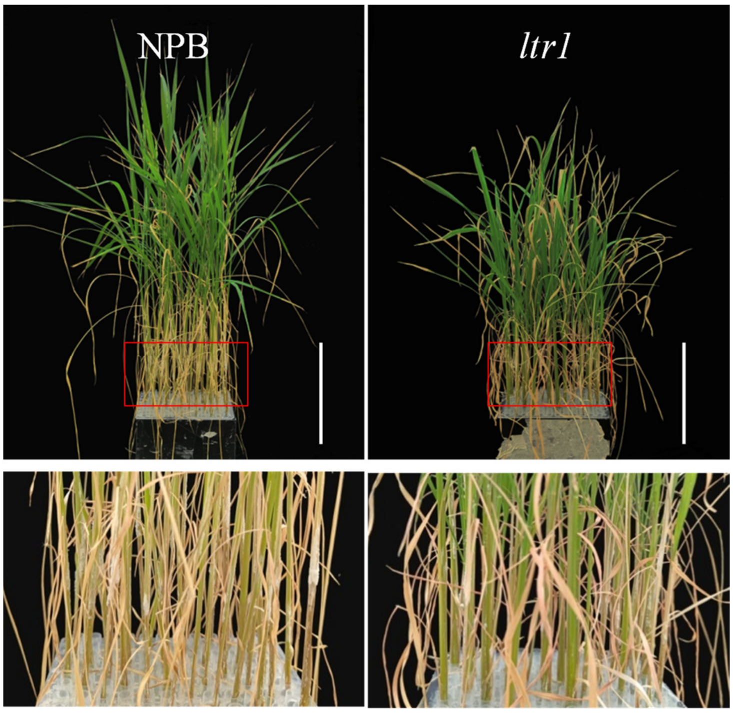

Figure S5. Plant morphology of NPB and *ltr1* under 100mM NaCl for 8 d, bar=11.5 cm.

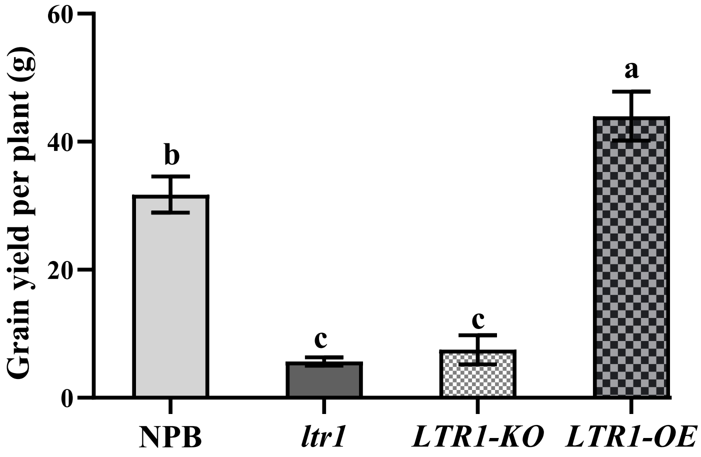

Figure S6. The grain yield per plant in lines of NPB, *ltr1*, *LTR1-KO*, and *LTR1-OE*. Data are given as means ± SD. Significant differences were determined by Duncan's new multiple range test and indicated with different lowercase letters ($p < 0.05$).
